# Multicentric Study to Assess *Helicobacter pylori* Incidence, Patient Reported Adverse Events, Compliance and Effectiveness, in Real-World Setting

**DOI:** 10.3390/ijerph191912847

**Published:** 2022-10-07

**Authors:** André Mesquita, Carlos Rocha-Castro, Daniela Guimarães, Joana Costa, Joana Soutinho, Tiago Taveira-Gomes

**Affiliations:** 1 USF Sanus Carandá, ACeS Cávado I-Braga, 4715-402 Braga, Portugal; 2 USF do Minho, ACeS Cávado I-Braga, 4715-402 Braga, Portugal; 3 USF Dr. Pelaez Carones, ACeS Cávado I-Braga, 4715-402 Braga, Portugal; 4 USF Gualtar, ACeS Cávado I-Braga, 4710-078 Braga, Portugal; 5 USF MaxiSaúde, ACeS Cávado I-Braga, 4700-036 Braga, Portugal; 6 MTG Research and Development Lab, 4200-604 Porto, Portugal; 7 Department of Community Medicine, Information and Decision in Health, Faculty of Medicine, University of Porto, 4050-313 Porto, Portugal; 8 Center for Health Technology and Services Research (CINTESIS), 4200-450 Porto, Portugal; 9 Faculty of Health Sciences, University Fernando Pessoa (FCS-UFP), 4249-004 Porto, Portugal

**Keywords:** *Helicobacter pylori* eradication, quadruple therapy with bismuth, effectiveness, compliance, adverse events

## Abstract

*Helicobacter pylori* (* H. pylori*) plays an important role in chronic gastritis and globally it is estimated to be present in half of the world’s population. In Portugal, prevalence reaches 85% and its eradication is recommended using quadruple antibiotic therapy, with or without bismuth. We intended to characterize the prescribed treatments evaluating effectiveness, adverse outcomes and compliance in a real-world setting in a primary care unit. A prospective multicenter observational cohort study was developed in five primary care units of Braga, Portugal. Patients diagnosed with *H. pylori* infection from August 2021 to January 2022 were included. Data were collected by interview (3 weeks after treatment) and review of medical records. Comparison between two groups of treatment and multivariable analysis was conducted. We estimated 13.4 cases per 1000 adults/year from 185 diagnoses. Therapy with bismuth was the most prescribed (83.8%) with a 96.7% eradication rate. There were no significant differences between treatments. Adverse events were reported in 73.8% of inquiries and female patients were associated with higher reports of nausea (*p* = 0.03) and metallic taste (*p* = 0.02). Both eradication schemes were effective and secure. The higher rate of adverse outcomes should be validated but it could influence the debate concerning treating all patients, especially in low gastric cancer-prevalence regions.

## 1. Introduction

*Helicobacter pylori* infection plays an important role in the development of chronic gastritis, in colonized individuals. Its transmission occurs by person-to-person dissemination and some of the risk factors include lower socio-economic income, numerous families, bed sharing and lack of piped water. Most infections are acquired during childhood [1]. This infection can be detected in around 50% of the world population, more frequently in developing countries, where its prevalence can reach 80%. Higher prevalence can be seen in Africa (79.1%), Latin America and the Caribbean (63.4%) and Asia (54.7%). On the other hand, North America (37.1%) and Oceania (24.4%) present lower prevalence of *H. pylori* infection. In Europe, prevalence of infection is not equal for all countries and it has a wide range of variety, remaining high in countries like Spain and Portugal, despite the improvement of sanitary and economic conditions [2]. In fact, Portugal is one of the countries with the highest prevalence of *H. pylori* infection worldwide. Data indicate infection rates ranging between 31.6% to 66.25%, with incidence rates between 4.1 and 11.6/100 people-year. The prevalence in adults appears to be above 60%, reaching 85% in some areas, such as in the north of Portugal [3,4].

The *H. pylori* infection can be presented with dyspeptic symptoms, but many individuals are asymptomatic and, in the long term, if not eradicated, *H. pylori* can lead to peptic ulcer disease, atrophic gastritis, gastric adenocarcinoma and MALT lymphoma. New data show that *H. pylori* infection always causes gastritis, irrespective of symptoms or complications, but being a major risk factor to gastric cancer, it is implicated in 90% of this neoplasm [5]. *H. pylori* eradication can prevent disease progression and long-term complications. The infection can be diagnosed by performing upper digestive endoscopy with gastric biopsies. In low *H. pylori* prevalence areas, a urease respiratory test is recommended [5].

*H. pylori* infection was recently considered a nosological entity itself in the new International Classification of Disease 11th Revision (ICD 11), which should imply treatment of all the infected patients. However, eradication recommendations are still in debate. Malfertheiner P. et al. reached a consensus in which treatment should be a priority in regions with intermediate or high gastric cancer incidence, and in populations older than 50 years old [5]. One of the main challenges of treatment is the increased *H. pylori* antibiotics resistance seen worldwide, which requires a constant update in pharmacological approaches. The use of triple therapy is not recommended in areas where clarithromycin resistance is higher than 15%. In this case, it is advised to use quadruple therapy with bismuth and, if not available, quadruple therapy without bismuth [5]. When clarithromycin resistance is lower than 15%, triple therapy with clarithromycin or bismuth quadruple therapy can be used, if proven effective locally [5].

In Portugal, antibiotic resistance is a known problem: the primary resistance rate to clarithromycin is 30% and to metronidazole it ranges between 10 and 30%. It is estimated that 5% of Portuguese adults present a dual resistance to both antibiotics [3]. Given this data, the first choice treatment in *H. pylori* eradication in Portugal should be quadruple therapy, with or without bismuth. It is recommended that the duration of treatment should last 14 days, unless the 10-day treatment is proven effective locally [5]. The effectiveness of treatments available in Portugal can differ. The bismuth quadruple therapy, containing tetracycline, metronidazole, bismuth and a proton pump inhibitor (PPI), during 10 days, obtained an eradication rate greater than 90%. The quadruple therapy without bismuth, containing amoxicillin plus metronidazole, clarithromycin and PPI, for 14 days, achieved an eradication rate between 86 and 91% [6].

One of the factors that can influence the compliance and treatment effectiveness is the presence of side effects. The reported side effects seemed to be of a similar sort and frequency between different therapies [7,8,9]. Regarding bismuth quadruple therapy, available in three-in-one single capsules, at least one adverse effect was reported by 29% of the patients. The most common were nausea in 9.5%, diarrhea in 8%, fatigue in 6.5% and metallic taste, dyspepsia and abdominal pain, each in 5% of patients. The presence of side effects caused treatment cessation in 2% of cases [10]. Other therapies, such as clarithromycin and metronidazole, can cause taste impairment or metallic taste. With metronidazole, gastrointestinal disturbances are also frequent. Treatment with levofloxacin can also lead to gastrointestinal disturbances, or to more severe side effects such as prolonged QT interval, tendinopathy with tendinous rupture and neurological dysfunctions (usually mild headache and dizziness and rarely severe delirium, agitation and disorientation) [7].

Another compliance-related factor is the cost of treatment. Although bismuth quadruple therapy is one of the most effective treatments, it is also the most expensive, with 10-day treatment costing 54.95€. The quadruple therapy without bismuth costs around 33€ for 14 days of treatment [11]. Furthermore, the strict posology and the higher number of pills that are required for the therapy with bismuth can have a negative effect on compliance (three pills taken four times a day, while therapy without bismuth needs three pills taken two times a day).

After treatment, the eradication confirmation can be obtained with urease respiratory test or fecal antigen screening, both displaying high specificity and sensibility, with the upside of being non-invasive tests. Another valid choice is performing upper digestive endoscopy with gastric biopsies [1,5].

*H. pylori* infection is a major gastric cancer risk factor, whose incidence is rather high in Portugal, particularly in the Northern Portugal region [12]. Among oncologic diseases, gastric cancer has the third highest mortality rate, which makes *H. pylori* eradication essential.

The main goal of this study is to characterize the pharmacological treatments prescribed in *H. pylori* eradication, namely quadruple therapy with bismuth and quadruple therapy without bismuth, in different primary healthcare facilities of a Northern Portugal city and evaluate their effectiveness, adverse outcomes and compliance.

## 2. Materials and Methods

Study composition: Prospective observational cohort study, developed in five primary care units of Braga’s Group of Health Centers, in Portugal. The study included patients with *H. pylori* infection diagnosed between August 2021 and January 2022, from the five units and no exclusion criteria were applied.

Subjects’ recruitment: This was performed by all the doctors working in the several primary care units, whenever a new *H. pylori* infection diagnosis was made during the study period. All patients were admitted without an age limit. After obtaining the patient’s written informed consent for study participation, the unit investigator was notified. Around three weeks after the expected end date of the treatment, a telephone interview was conducted with all subjects to determine the degree of comprehension of the treatment, therapy compliance and the presence of the most common side effects, like nausea, vomiting, diarrhea, metallic taste, abdominal pain and cutaneous rash. To standardize the subject’s evaluation, the investigators created a script for the telephone interview. Compliance, prescription comprehension and subjective degree of difficulty in completing the scheme are subjective variables and raw data concerning them were collected with a scale of 1 to 5 (the higher the value, the higher the compliance, comprehension and difficulty, respectively). For difficulty, the scale was very easy, easy, medium, hard or very hard. In order to simplify the analysis, the scale for difficulty was later changed into a three category variable, in which the degrees 1 and 2 were grouped as “easy”, degree 3 remained as “medium difficulty” and degrees 4 and 5 were grouped as “hard”. Similarly the scales for compliance and comprehension were changed to binary variables (5 = full compliance/comprehension; 1–4 = poor compliance/poor comprehension). The remaining variables were collected by clinical process analysis. To ensure diagnosis and testing of *H. pylori* was conducted in accordance with the latest European guidelines, we cross referenced the information that was stated in the medical records with that given by the patient. In particular, an initial diagnosis was confirmed to be made by upper digestive endoscopy with gastric biopsies, urease respiratory test or stool antigen tests in all patients and the confirmation tests of eradication had to be conducted at least 4 weeks (preferentially after 8 weeks) after scheme completion. Additionally, during the telephonic interview, patients were alerted to avoid proton-bomb inhibitors for 1 to 2 weeks before the eradication confirmation test, to lower the chance of false-negative results.

Data analysis: Each investigator was assigned to collect the data from each primary care unit, which included demographic data, smoking status, social problems and a depressive or anxiety disorder as coded in medical reports, prescribed treatment, duration of treatment, type of eradication test, timing for its realization and result and type of infection (primary, reinfection or previous treatment resistance). The variables were chosen based on the influence that they could have on one of our primary outcomes: effectiveness, compliance or adverse events. Selection was based on previous studies or clinical common knowledge. For example, in 2006, Suzuki T. et al. proposed that smoking could be related to a lower eradication rate [13], while DiMatteo M. et al., in 2000, showed that depression could be seen as a risk factor for noncompliance with any medical treatment [14].

Statistical analysis: Data were analysed using the Statistical Package for the Social Sciences^®^ (Version 26.0, company IMB, Armonk, NY, USA). Quantitative variables were summarized as the mean and standard deviation. Qualitative variables were represented with their respective absolute and relative frequencies. The results were categorized in two groups, according to the used *H. pylori* eradication scheme: quadruple therapy with bismuth versus quadruple therapy without bismuth. The chi-square test was used to examine differences at baseline between these two groups. A logistic binary regression model was used to identify key predictors towards the outcomes of interest (eradication, adverse effects and compliance) for each of the schemes. The categorical variables in this model included age, sex, economic insufficiency, education status, adverse effects and subjective degree of difficulty. A *p*-value less than 0.05 was considered statistically significant.

## 3. Results

From August 2021 until January 2022, 185 cases of *H. pylori* infection diagnosis were collected from the five study centers. In this time, 27,466 adults were observed in a primary care setting, resulting in a calculated incidence rate of 13.4 cases per 1000 adults/year. The average age of the sample was 55.9 (+/−11.9) years and the majority were women (58.4%). 

From this pool of diagnoses, 160 were included in our study, as 25 patients were excluded (13 did not initiate the treatment that was prescribed, 1 did not have any treatment prescribed, 6 were not available to conduct the interview at the time of the study and 5 subjects represented a small group which was prescribed a triple therapy scheme, with or without levofloxacin). This way, we eliminated groups with small sample size and our primary analysis focused on quadruple therapy with or without bismuth, from a total of 160 patients. The demographic characteristics of this sample are described in Table 1.

### 3.1. Descriptive Results

The choice of the therapy scheme of *H. pylori* eradication was performed by the family physician of each patient, mostly in face-to-face observation (61.9%). More than 90% of patients were diagnosed with *H. pylori* infection for the first time, whereas the rest were cases of reinfection or resistance to the first line of treatment (7.5% and 0.6%, respectively). Quadruple therapy with bismuth was the most prescribed treatment (83.8%). The patients’ basal characteristics were similar in each group, and balance was achieved, except for the rate of anxiety/depressive disorder (more information in Table 2), significantly higher in the quadruple therapy without bismuth (*p* = 0.015).

Regarding effectiveness, we found four cases of treatment resistance. Quadruple therapy with bismuth had 96.7% *H. pylori* eradication rate while quadruple therapy without bismuth had 94.7% eradication rate and no significant differences were found between these two groups.

### 3.2. Adverse Events

Event rates for adverse outcomes were similar for both quadruple therapies, as no significant differences were found between the two cohorts (Table 3). Overall, at least one adverse outcome was reported in almost three quarters of the interviewed patients (73.8%), with metallic taste, nausea and abdominal pain being the most common (39.4%, 36.9% and 31.2%, respectively). In two patients, the prescribed treatment was suspended at the second day, both with bismuth quadruple therapy (1.5%).

### 3.3. Compliance

**Figure 1:**
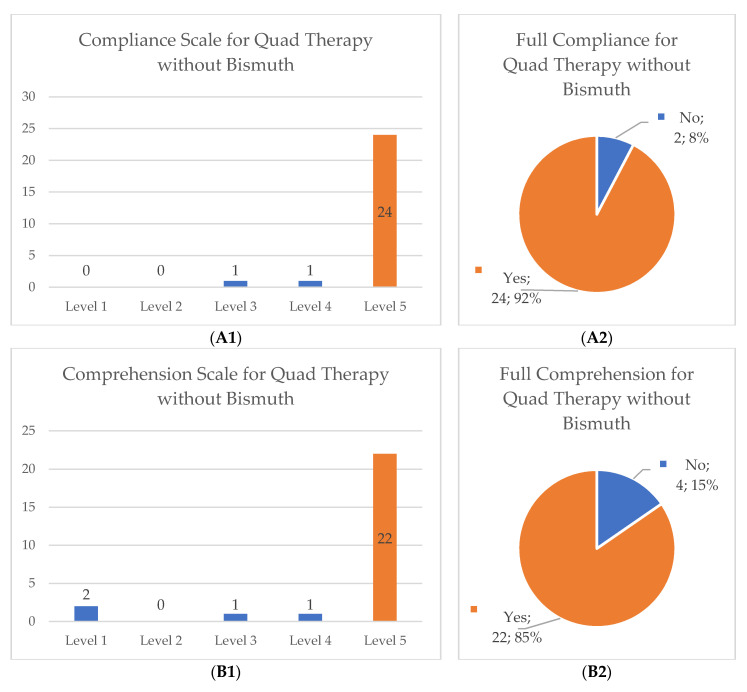

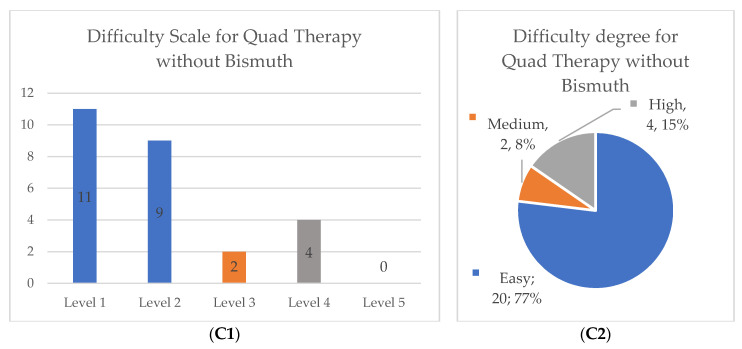
Distribution of data collected from compliance, comprehension and difficulty in Quadruple Therapy without Bismuth: the original scales (**A1**,**B1**,**C1**) and the new categorical organization of data (**A2**,**B2**,**C2**). Levels 1 to 5 represent a categorical scale used by the authors (a higher level corresponds to higher/full compliance, higher comprehension of prescribed posology of treatment and higher difficulty felt in completing the scheme).

After reviewing the collected data, we categorized the scales for compliance, comprehension of the prescribed scheme and scale of difficulty to eliminate unbalanced groups and to allow proper statistical analysis, particularly in the quadruple therapy without bismuth group (original distribution of data and its new categorization can be found in Figure 1). The compliance and comprehension scales were changed into two dichotomous categorical variables: number 5 equals full compliance or full comprehension and numbers 1 to 4 implied forgetting at least one pill or not fully comprehending the scheme. The difficulty degree was changed into a three category system: easy (numbers 1 and 2), moderate (number 3) and hard (numbers 4 and 5).

Full compliance of the treatment scheme was achieved in most cases (85.6%) as well as full comprehension of the treatment scheme (89.4%) without significant differences between the two major treatment groups. Regarding the degree of difficulty in completing the scheme, there was a higher distribution of participants who subjectively characterized the quadruple therapy with bismuth as hard (20.9% versus 15.4%) or as medium difficulty (20.9% versus 7.7%), but no significant differences were found between both cohorts (*p* = 0.16). More details in Table 4.

### 3.4. Multivariable Statistic

Multivariable logistic regression analysis was performed to find variables that could influence the *H. pylori* eradication rate, adverse outcome rates and compliance. Due to the low sample size for quadruple therapy without bismuth, this analysis was not conclusive and therefore the data were omitted.

**Figure 2:**
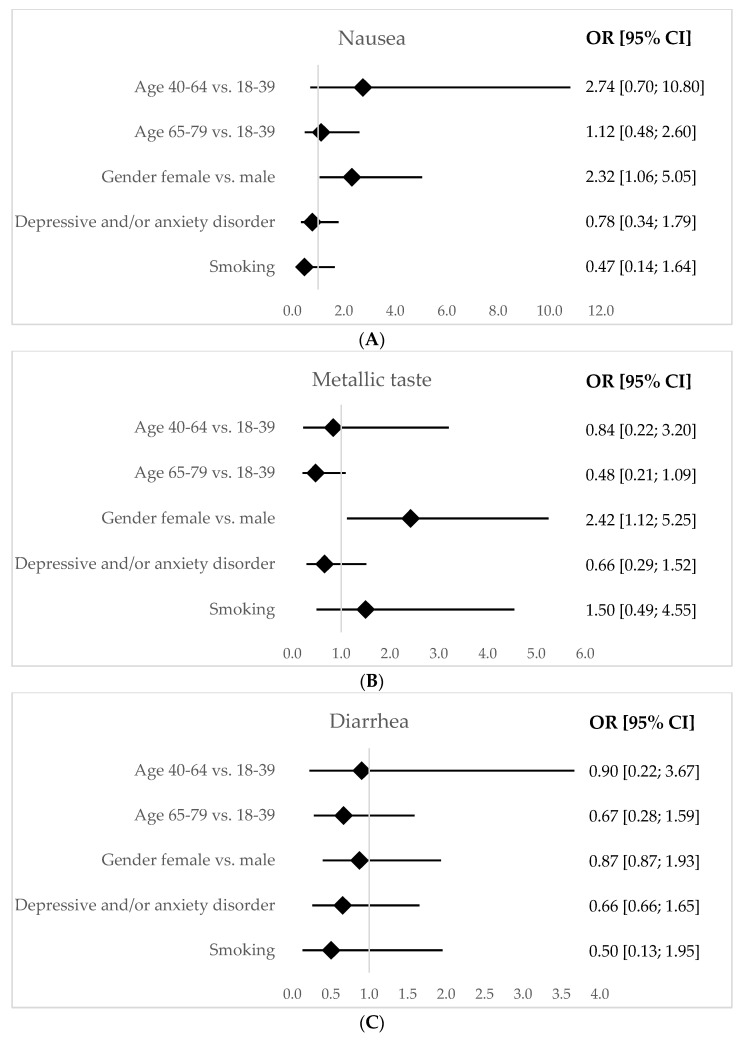

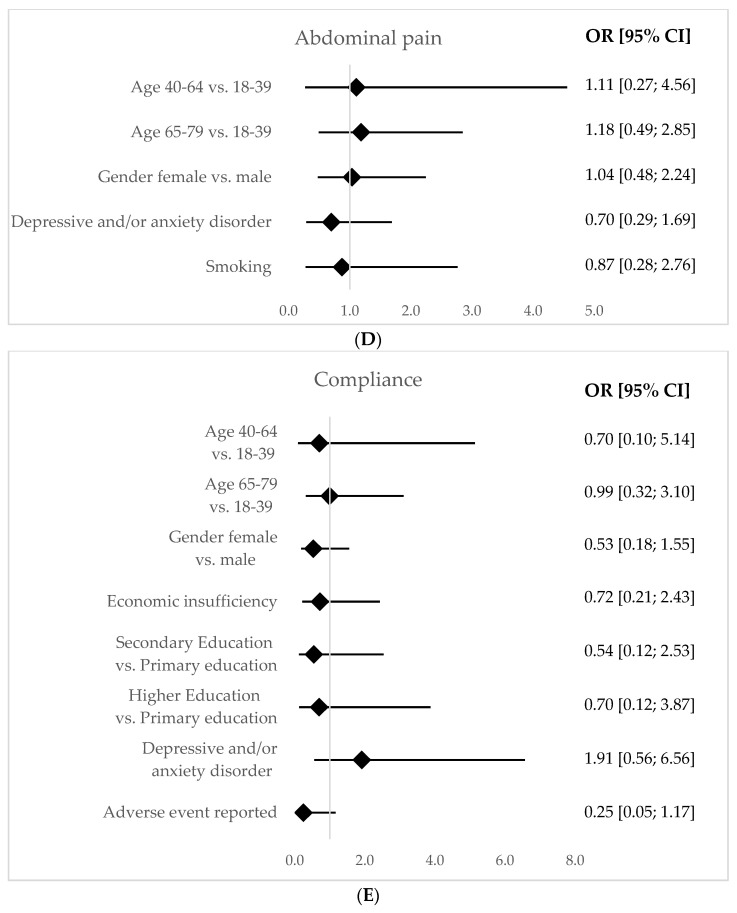
Forest plot charts for multivariable analysis of adverse outcomes and scheme compliance for quadruple therapy with bismuth: nausea (**A**), metallic taste (**B**), diarrhea (**C**), abdominal pain (**D**), scheme compliance (**E**). (CI—Confidence Interval; OR—Odds Ratio).

For the bismuth group, regarding adverse outcomes, we found that female patients reported more nausea (OR = 2.32; CI [1.5–5.6]; *p*-value = 0.03) and metallic taste (OR = 2.42; CI [1.12–5.25]; *p*-value = 0.02). The analysis for vomits and skin rash was not conclusive due to the low sample size (data omitted). All results are displayed in Figure 2. Additionally, we found that a higher degree of difficulty of 4 or 5 (in a subjective scale from 1 to 5) was associated with less compliance (OR = 0.47; CI [0.26–0.86]; *p*-value = 0.01).

## 4. Discussion

In this study we performed a broad evaluation of the patients with *H. pylori* infection and their treatment in a multicentric setting in Braga, Portugal, the first of its kind in this region, for a 6-month period. Acknowledged as one of the countries with the highest prevalence of *H. pylori* infection, our study showed an incidence rate similar to other studies in Portugal [3,4]. Prevalence was not assessed as there were no data available on how many patients were screened with endoscopy or other diagnosis methods during the study period.

We assessed effectiveness, adverse outcome ratio, compliance and subjective degree of difficulty for each therapy scheme, particularly quadruple therapy with or without bismuth. Regarding effectiveness, we found that both quadruple therapies achieved the accepted threshold of a 90% eradication rate for an optimal *H. pylori* treatment [15], in a real-world setting. Additionally, we evaluated further factors that could modulate effectiveness, but no variable appeared to significantly influence this outcome.

Concerning the difficulty degree, and even though no significant differences were found between groups, our study showed that the quadruple therapy with bismuth was reported as being more difficult to carry out. One of the reasons behind this finding might be related with the posology for treatment completion (higher number of pills and short interval between doses) as we were casually alerted by the participants themselves. We believe a new study with a higher number of patients and more balanced groups is needed.

Regarding the adverse outcome rate, nearly three out of four patients reported at least one event. Our real-world analysis showed a higher rate of nausea, diarrhea, abdominal pain and metallic taste than reported in previous studies [10] (almost four times higher). That said, Nyssen O. et al. [10] did not state the method how their adverse events were evaluated, as patient-reported or medical-evaluated sorts can highly influence the rate of these outcomes. Nonetheless, this result should be accounted for when choosing to implement the eradication scheme in asymptomatic patients with no gastric alterations at endoscopy, especially when the benefit of treatment is low even though international guidelines support the eradication of *H. pylori* infection in all patients in countries with high prevalence of infection, such as Portugal [5]. We found that the reports of different adverse outcomes were higher in female patients, but we cannot find any reasonable explanation for this result as it can be related to a number of variables that were not accounted for in this study. Besides that, these events were similar between the two major groups of treatment, and no severe adverse outcome was reported. It should also be noted that two cases of treatment interruption were found with quadruple therapy with bismuth, but due to the low sample size, no evaluation was made.

Given the high rate of antibiotic resistance [3,5] and high rate of adverse outcomes reported, the question of not treating all colonized patients can be supported. This matter is particularly important, since the most recent consensus does not make any categorical statement about expanding its recommendation to treat all colonized patients, specifically, of regions of low gastric cancer incidence [5]. That said, this study may add to the case that eradication in these latter regions should be reserved to symptomatic patients or to those with individual high risk of gastric cancer, such as family history of cancer.

For a hypothetical cost–benefit evaluation, since this was not the aim of this article, we can project that, because both therapies show a similar effect, quadruple therapy without bismuth has some economic advantages. This fact is also supported by INFARMED^®^ (Portugal’s national authority for pharmaceutical regulation), who in July of 2022 denied co-payment of the therapy with bismuth within the Portuguese National Health Service (SNS) funding [16].

Several limitations of our study can be noted such as the small sample groups and the non-randomized exposure that led to slightly unbalanced groups, particularly in patients with anxiety/depressive disorder background. With regard to the methodology used, as it is a real-world study, authors must admit that some variables cannot be fully managed, such as the therapeutical compliance of the proton-bomb inhibitor suspension before the eradication confirmation, that could lead to a small percentage of false-negatives, and thus overestimating the efficacy of the treatment schemes. It should also be mentioned that, as the patients needed to be referred by their family physician, some *H. pylori* infection cases might have been lost and the overall diagnosis incidence might be underestimated. Additionally, the study may be susceptible to a geographical bias since our findings are focused on the Portuguese population. However, further comparison shows similar outcomes in foreign studies such Lin L. et al., in the Chinese regions, that achieved a similar effectiveness rate for the quadruple therapy without bismuth [17] or Gravina AG. et al., in Italy, for the therapy with bismuth [18]. Other limitations include the relatively short assessment period of adverse events and the self-reported method which can overestimate the rate of these events. Nevertheless, this study was conducted in a real-world setting providing more useful evidence for clinical practice with freedom to select the treatment scheme and a high coverage of local patients. We also believe that the short interval between the telephonic interview and treatment completion allowed a more accurate assessment of the variables that were evaluated, particularly the subjective ones, and adverse outcome reports.

## 5. Conclusions

In summary, we developed a broad study of the *H. pylori* infection and its eradication treatment in the region of Braga, Portugal, in a real-world setting through primary care clinical practice. We found that both quadruple therapies with or without bismuth were effective and secure. Although overall adverse outcome rates were higher than in previous articles, there were no differences between the two groups and compliance was not affected. Therefore, the choice of eradication scheme could attend to the fact that quadruple therapy with bismuth has shown tendencies of being subjectively more difficult and that there is a 22 euro gap between Pylera^®^, the only quadruple therapy with bismuth available in Portugal, and the cheaper quadruple therapy without bismuth composed of three antibiotics (amoxicillin, metronidazole and clarithromycin). That said, it is still important to note that similar studies with a stronger sample size should be conducted to verify the higher adverse outcome rate reported in this study and to determine the long-term adverse outcomes that could unbalance the scale of risk/benefit in treating specially patients in low gastric cancer incidence regions.

## Figures and Tables

**Table 1:** Patients’ characteristics.

	DIAGNOSED *H. PYLORI* CASES (*N* = 160)
	*N* (%)
SEX	
FEMALE	95 (59.4)
AGE	
MEAN ± SD	56.2 ± 11.8
18–39 YEARS	15 (9.4)
40–64 YEARS	104 (65.0)
65–79 YEARS	41 (25.6)
ECONOMIC INSUFICIENCY	
YES	35 (21.9)
EDUCATION STATUS	
4TH TO 9TH GRADE	99 (61.9)
10TH TO 12TH GRADE	31 (19.4)
UNIVERSITY EDUCATION	30 (18.8)
SMOKING STATUS	
NON-SMOKER	137 (85.6)
CURRENT SMOKER	23 (14.4)
SOCIAL PROBLEMS	
YES	7 (4.4)
ANXIETY/DEPRESSIVE DISORDER	
YES	48 (30.0)
PRESCRIBED TREATMENT	
PPI + AMOX + CLAR + MET^1^ ^a^	26 (16.3)
PPI + BIS + MET^2^ + TET	134 (83.7)

Abbreviations: *N*—number of cases; SD—standard deviation; PPI—Proton pump inhibitor; Amox—Amoxicilin 1 g; Clar—Clarithromycin 500 mg; Met^1^—Metronidazole 500 mg: Bis—Bismuth 140 mg; Met^2^—Metronidazole 125 mg; Tet—Tetracycline 125 mg. ^a^ one patient in this group completed 2-day therapy with “PPI + Bis + Met^2^ + Tet” and then switched to “PPI + Amox + Clar + Met”.

**Table 2:** Characteristics of the patients in each treatment group.

	QUADRUPLE THERAPY WITH BISMUTH (*N* = 134)	QUADRUPLE THERAPY WITHOUT BISMUTH (*N* = 26) ^a^	*P*-VALUE
	*N* (%)	*N* (%)	
SEX			0.849
FEMALE	80 (59.7)	15 (57.7)	
AGE			0.879
MEAN ± SD	56.1 ± 12.9	56.3 ± 12.6	
18–39 YEARS	13 (9.7)	2 (7.7)	
40–64 YEARS	86 (64.2)	18 (69.2	
65–79 YEARS	35 (26.1)	6 (23.1)	
ECONOMIC INSUFICIENCY			0.231
YES	27 (20.1)	8 (30.8)	
EDUCATION STATUS			0.754
4TH TO 9TH GRADE	83 (61.9)	16 (61.5)	
10TH TO 12TH GRADE	27 (20.1)	4 (15.4)	
UNIVERSITY EDUCATION	24 (17.9)	6 (23.1)	
SMOKING STATUS			0.167
NON-SMOKER	117 (87.3)	20 (76.9)	
CURRENT SMOKER	17 (12.7)	6 (23.1)	
SOCIAL PROBLEMS			0.366
YES	5 (3.7)	2 (7.7)	
ANXIETY/ DEPRESSIVE DISORDER			0.015
YES	35 (26.1)	13 (50.0)	
TYPE OF CONSULTATION			0.399
FACE-TO-FACE	81 (60.4)	18 (69.2)	
NON-FACE-TO-FACE	53 (39.6)	8 (30.8)	
TYPE OF INFECTION			0.058
PRIMARY	123 (91.8)	24 (92.3)	
REINFECTION	11 (8.2)	1 (3.8)	
PREVIOUS TREATMENT RESISTANCE	0	1 (3.8)	

Abbreviations: *N*—number of cases; SD—standard deviation ^a^ one patient in this group completed 2-day therapy with bismuth and then switched to the group without bismuth.

**Table 3:** Reported adverse events.

	QUADRUPLE THERAPY WITH BISMUTH (*N* = 134)	QUADRUPLE THERAPY WITHOUT BISMUTH (*N* = 26)	*P*-VALUE
	*N* (%)	*N* (%)	
ADVERSE EFECTS			
NAUSEA	52 (38.8)	7 (26.9%)	0.250
VOMITING	10 (7.5)	2 (7.7%)	0.968
ABDOMINAL PAIN	42 (31.3)	8 (30.8)	0.954
METALLIC TASTE	54 (40.3)	9 (34.6)	0.587
DIARRHOEA	38 (28.4)	8 (30.8)	0.804
RASH	6 (4.5)	2 (7.7)	0.491
AT LEAST ONE AE	98 (73.1)	20 (76.9)	0.688

Abbreviations: *N*—number of cases; AE—adverse effects.

**Table 4:** Compliance, degree of difficulty and comprehension of the treatment.

	QUADRUPLE THERAPY WITH BISMUTH (*N* = 134)	QUADRUPLE THERAPY WITHOUT BISMUTH (*N* = 26)	*P*-VALUE
	*N* (%)	*N* (%)	
COMPLIANCE			0.289
YES (5)	113 (84.3)	24 (92.3)	
NO (<5)	21 (15.7)	2 (7.7)	
COMPREHENSION OF TREATMENT			0.389
YES (5)	121 (90.3)	22 (84.6)	
NO (<5)	13 (9.7)	4 (15.4)	
DIFFICULTY			0.165
EASY (1, 2)	78 (58.1)	20 (76.9)	
MEDIUM (3)	28 (20.9)	2 (7.7)	
HARD (4, 5)	28 (20.9)	4 (15.4)	

Abbreviations: *N*—number of cases.

## Data Availability

Not applicable.
